# High‐Density Topological Defect Array by Two‐Step Interference Photoalignment

**DOI:** 10.1002/adma.72607

**Published:** 2026-02-19

**Authors:** Sunqian Liu, Inge Nys, Kristiaan Neyts

**Affiliations:** ^1^ Liquid Crystals and Photonics Group Department of Electronics and Information Systems Ghent University Ghent Belgium; ^2^ State Key Laboratory of Displays and Opto‐Electronics Department of Electronic and Computer Engineering Hong Kong University of Science and Technology Hong Kong China

**Keywords:** grating, interference illumination, liquid crystal, photoalignment, topological defect

## Abstract

With nematic liquid crystal (LC) topological defects can be created that are important for the generation of laser beams with orbital angular momentum. Arrays of defects have been realized through photoalignment, by projecting images of a spatial light modulator or digital mirror device. However, such pixel‐based approaches seriously limit the achievable density of defects. Here, we propose a scalable method based on two‐step interference illumination to achieve a much higher density. In the first step, a rotating director pattern is generated by the interference of two circularly polarized beams. In the second step, amplitude modulation by two‐beam interference is used to rewrite the pattern and introduce the defects. By adjusting the illumination doses and angles of incidence in both steps, 2D defect patterns are obtained, with spacing down to 1.25 micrometer. The resulting disclination lines are analyzed, and the proposed structure is supported by numerical simulations. For the first time, a high defect density defect grid is obtained through a scalable two‐step photoalignment procedure. This approach enables optical components with large‐angle diffraction and bridges the gap between LC topological optics and metasurfaces.

## Graphical abstract

High density arrays of topological defects in nematic liquid crystal are realized by two‐step interference photoalignment, with a minimal distance of 1.25 micrometer and disclination lines connecting defects on opposing substrates. Such layers can be used as reconfigurable templates for photonic devices to achieve large angle diffraction, beam shaping with large numerical aperture, or bistable smart windows.

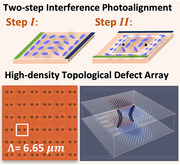

## 1 Introduction

Nematic liquid crystal (LC) is a self‐organizing liquid with uniaxial symmetry, used in displays, smart windows, electro‐optic switches, and soft robots [1, 2, 3]. Recently, there has been an increased interest in line‐shaped topological defects where the director is discontinuous [4]. These disclination lines are topologically protected and form either a closed loop or terminate at the boundary of the LC volume. Inversely, when the director pattern at the surface contains a point where the director is discontinuous, one or more disclination lines may originate from this point. The creation of disclination lines based on a defect pattern at the surface has been studied in detail [5, 6, 7, 8, 9, 10, 11, 12, 13, 14]. There are many applications in which topological defects can be used, for example, in diffractive components with multi‐stability, which is difficult to obtain in defect‐free devices [15, 16, 17, 18].

Photoalignment is a well‐known technique to control LC alignment by illuminating a photosensitive (for example, azo‐dye containing) layer with polarized blue or UV light. Interference illumination with two beams of equal intensity and opposite circular polarization can achieve a simple periodic rotating pattern with a short period [19, 20, 21, 22]. Projecting the image of a spatial light modulator (SLM), after translating the phase retardation into the azimuthal angle of linear polarization, yields arbitrary alignment patterns [14, 23, 24]. A similar result can be obtained by projecting linearly polarized light transmitted through a mask [25, 26] or reflected from a digital mirror device [27, 28]. In both cases, the resolution is limited by the projection system, and the number of pixels is determined by the modulator. Finally, there is the method of direct writing, in which a narrow laser beam with variable linear polarization is scanned over the substrate [29, 30, 31, 32]. This method can suffer from line‐to‐line scanning errors, and it is slow when features with a small diameter are required. In principle high resolution 2D alignment grids can also be realized by an interference setup with three or four laser beams, but in this case, the depth of focusing is very small [33, 34, 35].

The creation of defects in the alignment pattern has been demonstrated by many groups, but the distance between defects is always 20 µm or more [8, 9, 11, 23, 36, 37, 38], except when a nanohole mask realized by e‐beam lithography is used (distance 4 µm) [36]. In this work, we suggest a low‐cost scalable approach to create a periodic grid of surface defect points with distance almost down to one micrometer. By using a two‐step interference illumination procedure with well‐designed polarization patterns, we achieve a 2D alignment pattern with a periodic array of half‐integer defects. After filling the cell with nematic LC we observe the transmission image with the polarization microscope. The analysis of the resulting images is achieved by combining the previously developed theoretical model for multi‐step photoalignment illumination [39] with numerical simulations for the LC director in the volume and optical simulations based on a beam propagation method. Moreover, the diffraction properties of the produced defect grids are measured to highlight the application potential in large angle diffractive optical components.

## 2 Defect Pattern Creation Based on Two‐Step Interference Photoalignment

**FIGURE 1:**
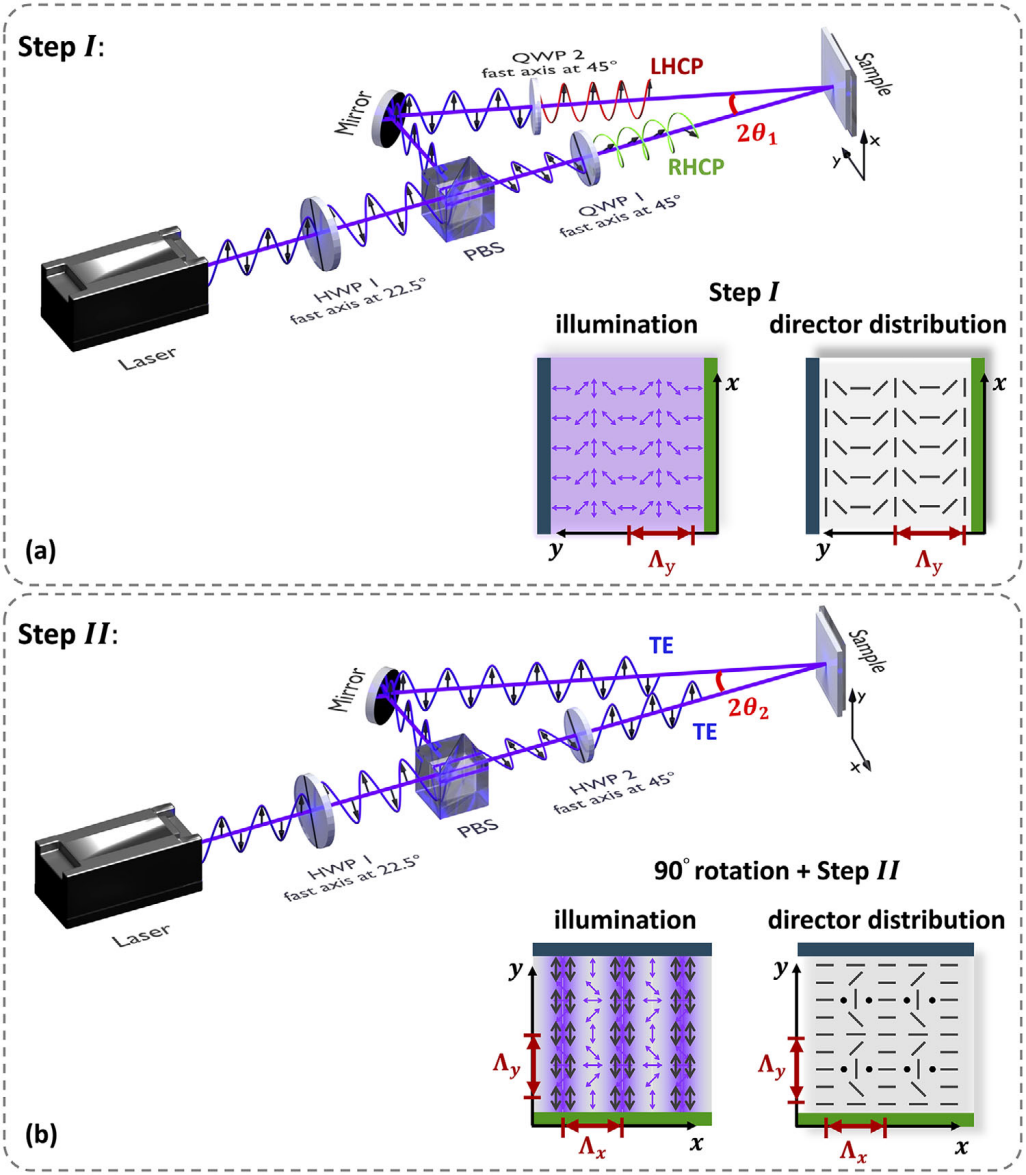
Setup for two‐step interference photopatterning. HWP 1 controls the relative intensities of the two beams after the PBS. (a) Step I: illumination with right‐ and left‐handed circularly polarized light by setting the fast‐axis of the QWPs at ± 45° to obtain an interference pattern. The polarization/intensity of the illumination and the resulting director distribution are shown separately. (b) Step II: illumination with two linearly polarized TE (along the *y*‐axis) beams with equal power for intensity modulation. The intensity modulated interference pattern with polarization along the *y*‐axis, overlayed with the polarization pattern obtained in step I, and the resulting director distribution (with defects) are shown separately. The period of the alignment pattern in both steps is given by $\Lambda =\frac{\lambda }{2\sin\theta }$, with θ the half opening angle between the interfering beams.

Here, we propose a two‐step interference photoalignment procedure to realize an array of closely spaced alignment defects with half‐integer strength. For the fabrication of the LC cell, we use a standard procedure, based on two pretreated glass substrates coated by a brilliant yellow (BY, Sigma–Aldrich) photoalignment layer and glued together by a glue that contains spherical spacers with a diameter 1.6 µm, according to the Experimental Section. The cell is placed in the optical set‐up and illuminated with a two‐step UV interference exposure. The set‐up for the two illumination steps is illustrated in Figure 1 and explained in more detail in the Experimental Section. In the first step (Figure 1a), a rotating director pattern is realized with variation along the *y*‐axis (due to polarization modulation in the interference plane). To achieve this pattern, two quarter‐wave plates (QWP) are used to achieve right‐handed (RHCP) and left‐handed circular polarization (LHCP), respectively. Before the second illumination step (Figure 1b), the cell is rotated over 90° to achieve an interference pattern with intensity variations along the *x*‐axis. The two QWPs are removed to obtain interference between two beams with identical intensity and TE polarization. The second illumination partially overwrites the alignment achieved after the first illumination, in particular in the antinodes of the interference pattern, resulting in a 2D alignment pattern. The period of the patterns in the *x*‐ and *y*‐direction, Λ_*x*_ and Λ_*y*_ depend on the angle θ between the interfering beams and the substrate normal, according to $\Lambda_{y}=\frac{\lambda }{2\sin\theta_{1}}$ and $\Lambda_{x}=\frac{\lambda }{2\sin\theta_{2}}$. After the two‐step interference photoalignment procedure, the nematic LC E7 is filled into the cell above the isotropic temperature, and the assembly is slowly cooled to room temperature.

According to the previously proposed double illumination theory [39], the resulting azimuthal angle Φ between the easy alignment axis and the *x*‐axis, is given by the following equation:

(1) $$\begin{array}{c} \tan2\Phi \left(x,y\right)=\frac{-sin2\varphi_{2}\left[1-\exp\left(-aD_{2}\right)\right]-sin2\varphi_{1}\left[1-\exp\left(-aD_{1}\right)\right]\exp\left(-aD_{2}\right)}{-cos2\varphi_{2}\left[1-\exp\left(-aD_{2}\right)\right]-cos2\varphi_{1}\left[1-\exp\left(-aD_{1}\right)\right]\exp\left(-aD_{2}\right)} \end{array}$$

With φ_1_ and φ_2_ the azimuthal angles of the linearly polarized illumination in step I and step II, *D*_1_ and *D*_2_ (mJ cm^−2^) the illumination doses in step I and step II, respectively, and *a* (cm^2^ mJ^−1^) a sensitivity parameter that depends on the photoalignment material and the illumination wavelength.

For the illumination steps used here, with interference between RHCP and LHCP in step I and two TE polarizations in step II, we obtain:

$$\varphi_{1}\left(y\right)=\frac{\pi }{2}-\frac{\pi }{\Lambda_{y}}y;\varphi_{2}=\frac{\pi }{2};$$

(2) $$\begin{array}{c} D_{2}\left(x\right)=2D_{2avg}\cdot cos^{2}\left(\frac{\pi }{\Lambda_{x}}x\right) \end{array}$$

The azimuthal angle of the easy alignment axis Φ resulting from the two‐step photoalignment is determined by:

(3) $$\begin{array}{c} \tan2\Phi \left(x,y\right)=\frac{-sin\left(\frac{2\pi }{\Lambda_{y}}y\right)}{cos\left(\frac{2\pi }{\Lambda_{y}}y\right)+\frac{\exp\left(aD_{2}\left(x\right)\right)-1}{1-exp\left(-aD_{1}\right)}}\; \end{array}$$

Defects in the surface anchoring pattern occur when both the numerator and the denominator become equal to zero, and the illumination with perpendicular polarization in step two exactly compensates for the illumination effect in step one. This occurs when *y* is an uneven multiple of Λ_*y*_/2, and for the following condition for *x*:

(4) $$\begin{array}{c} exp\left(aD_{2}\left(x\right)\right)-1=1-exp\left(-aD_{1}\right) \end{array}$$

This condition can be satisfied when the average intensity of the second illumination is sufficiently strong:

(5) $$\begin{array}{c} D_{2avg}>\frac{1}{2a}\ln\left(2-exp\left(-aD_{1}\right)\right) \end{array}$$

In this case, there are two defects in every period Λ_*x*_, one with $s=+\frac{1}{2}$ and one with $s=-\frac{1}{2}$. The distance *d_surface_* between the two surface defects in the *x*‐direction (as illustrated in Figure 4) is given by:

(6) $$\begin{array}{c} d_{sur\;f\;ace}=\frac{2\Lambda_{x}}{\pi }\arcsin\sqrt{\frac{\ln\left(2-\exp\left(-aD_{1}\right)\right)}{2aD_{2avg}}} \end{array}$$

## 3 Experimental Results

**FIGURE 2:**
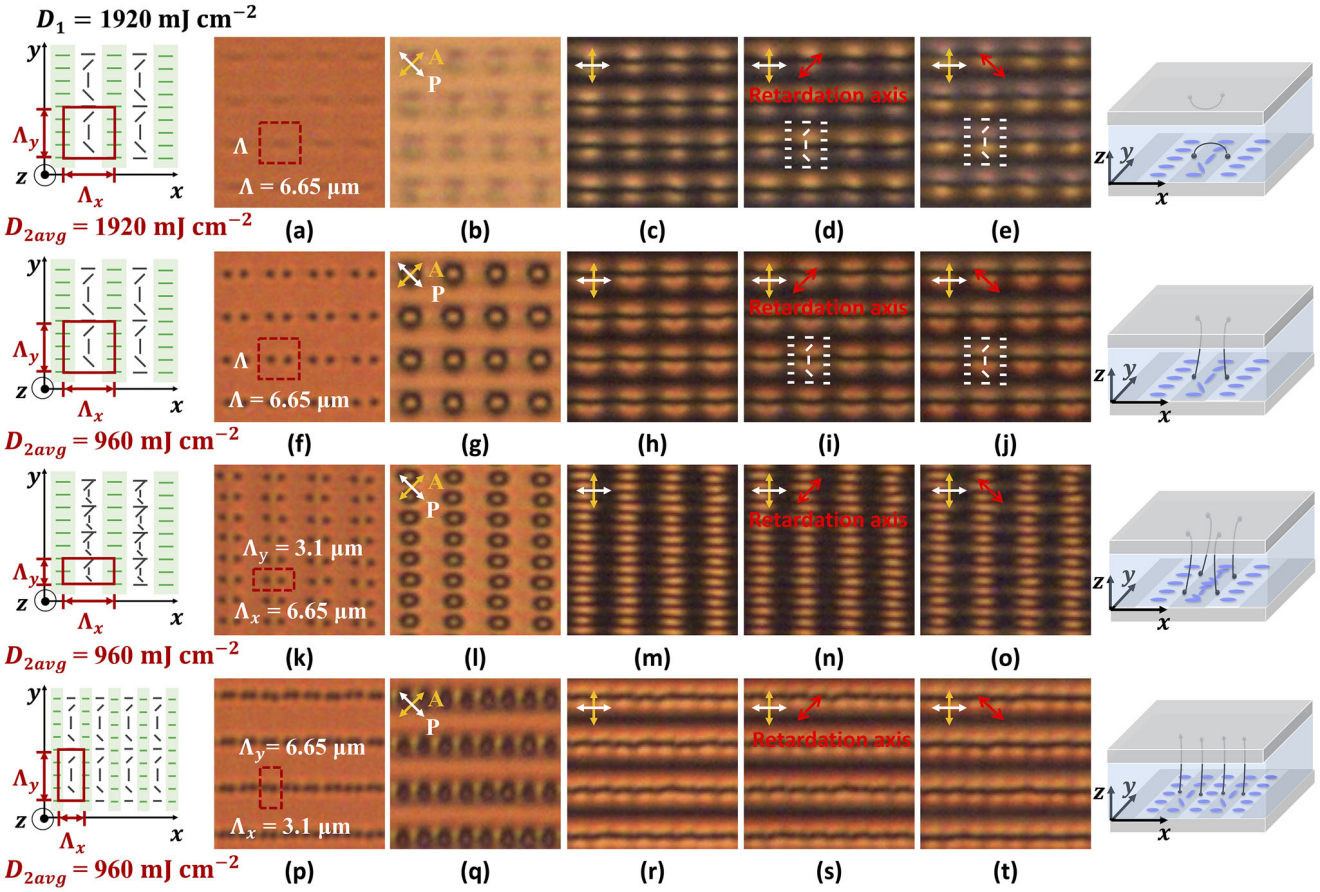
Microscopy images of 2D patterned defects in cells with thickness *t* around 1.4 µm. The illumination dose in step I was fixed at 1920 mJ cm^−2^, while in step II the illumination dose was 1920 mJ cm^−2^ in (a–e), and 960 mJ cm^−2^ in (f–t). The period of the photoalignment pattern along the *x*‐ and *y*‐axis is Λ_*x*_ = Λ_*y*_ = 6.65 µm, except in (k–o) where Λ_*y*_ = 3.1 µm, and in (p–t), where Λ_*x*_ = 3.1 µm. The diagrams on the left and right sides of the images illustrate, respectively the alignment patterns on the substrate and the resulting disclination lines.

Figure 2 shows transmission microscopy images for cells with a 2D defect pattern at the substrates and a cell thickness *t* around 1.4 µm. The periods along the *y*‐axis Λ_y_ and along the *x*‐ axis Λ_*x*_ are determined by the angles θ_1_ and θ_2_ in the interference setup in step I and step II, respectively. For the images shown in Figure 2a–j, the period is 6.65 µm in both directions, while a smaller period of 3.1 µm in the *y*‐ or *x*‐direction is used in Figure 2k–o and respectively Figure 2p–t. The illumination dose in step I *D*_1_ = 1920 mJ cm^−2^ is obtained by multiplying the intensity of 64 mW cm^−2^ with a duration of 30 s.

For the results shown in Figure 2a–e, the illumination in step II has an average dose *D*_2*avg*_ = 1920 mJ cm^−2^. After filling the cell with LC, the resulting image under the microscope without polarizers shows an array of short black/gray lines oriented along the *x*‐axis (Figure 2a). When the second dose is decreased to 960 mJ cm^−2^, an array of pairs of black dots appears, as shown in Figure 2f. The same behavior, with pairs of black dots, is seen for the configurations with the same illumination dose in the second step, but with other dimensions (Λ_*x*_, Λ_*y*_) for the anchoring pattern (Figure 2k,p). Black lines and dots in the images without polarizers indicate that unpolarized light is not transmitted through the structure but scattered due to fast variations in the director alignment. The short dark lines in Figure 2a correspond to disinclination lines that are connecting defect points on the same substrate. The dark dots can be identified as disclination lines that connect defects from the bottom substrate to the top substrate (as will be confirmed later). The positions of the defect points deviate slightly from an ideal grid, which may be due to imperfections in the alignment layer or in the interference illumination procedure.

Because the LC material has anisotropic optical properties, the director alignment can be indirectly evaluated by observing the samples with a microscope between crossed polarizers. For 45° orientation of the crossed polarizers (with respect to the *x*‐ and *y*‐axis), an array of black rings in a bright background can be seen in Figure 2g,l,q. Another configuration, with less pronounced dark features, is observed in Figure 2b. When rotating the crossed polarizers so that they are aligned along the *x*‐ and *y*‐axis, the background becomes dark, and in each unit cell, two bright regions become visible (see Figure 2c,h,m). To further identify the rotating director alignment along the *y*‐axis, a 37 nm retardation plate is added in the polarization optical microscope (POM) with its slow axis oriented at + 45°, as shown in Figure 2d,i,n,s. The upper part of the disc/the top arch undergoes a small color shift toward longer wavelengths, while the lower part shifts toward shorter wavelengths. The situation inverses when the retarder is at − 45°, as shown in Figure 2e,j,o,t. This indicates that LC director rotates clockwise along the + *y*‐axis (as indicated by white lines in Figure 2d,e,i,j).

To demonstrate the versatility of the photoalignment method, the defect patterns have also been experimentally realized for a smaller alignment period in the *x*‐ or *y*‐dimension, equal to 3.1 µm, by increasing the angle of incidence between the two laser beams. The illumination dose in the second step was fixed in these cases to *D*_2*avg*_ = 960 mJ cm^−2^ to obtain vertical disclination lines, connecting the top and bottom substrate. Microscopy images are shown in Figure 2k–t, demonstrating a large defect density. It is clear that when the pattern period along the *x*‐ axis is Λ_*x*_ = 3.1 µm shown in Figure 2p, neighboring defects along the *x*‐ axis are very close to each other, at a distance of about 1 µm.

**FIGURE 3:**
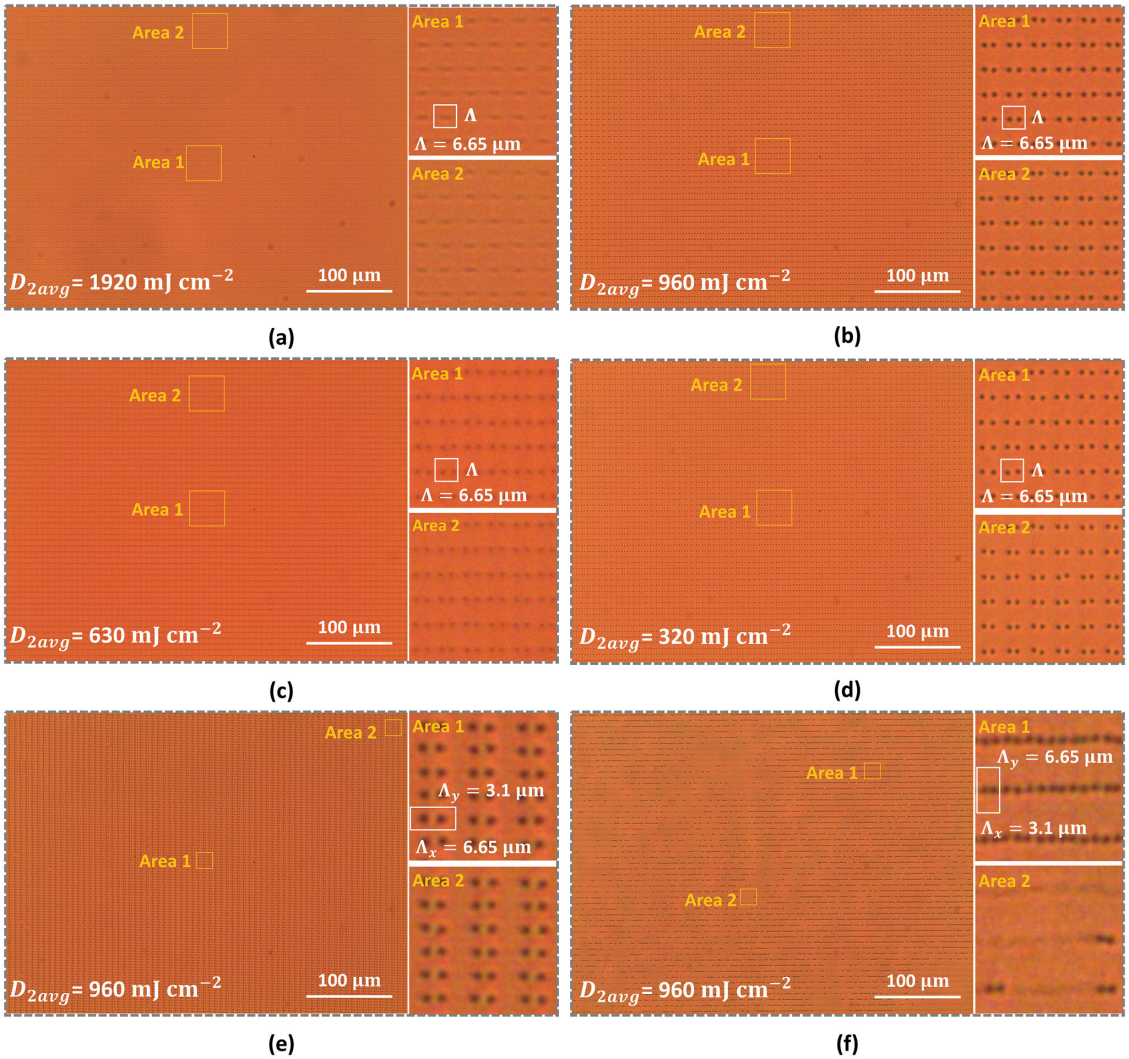
Microscopy images of defect patterns over a larger area, illustrating the homogeneity, for different doses *D*_2*avg*_ and the pattern period. Photoalignment patterns with Λ_*x*_ = Λ_*y*_ = 6.65 µm, and with *D*_2*avg*_ equal to 1920 mJ cm^−2^ in (a), 960 mJ cm^−2^ in (b), 630 mJ cm^−2^ in (c), and 320 mJ cm^−2^ in (d). Photoalignment pattern with Λ_*x*_ = 6.65 µm and Λ_*y*_ = 3.1 µm in (e) and Λ_*x*_ = 3.1 µm and Λ_*y*_ = 6.65 µm in (f).

As can be seen in Figure 3, the defect patterns can, in most cases, be generated over a relatively large area. Microscopy images without polarizers are shown for six different photoalignment patterns, for an area that covers several tens of defects in the *x*‐ and *y*‐direction. Three different photoalignment patterns have Λ_*x*_ = Λ_*y*_ = 6.65 µm and *D*_2*avg*_, respectively equal to 1920 mJ cm^−2^ (Figure 3a), 960 mJ cm^−2^ (Figure 3b), 630 mJ cm^−2^ (Figure 3c), and 320 mJ cm^−2^ (Figure 3d). One photoalignment pattern has Λ_*x*_ = 6.65 µm and Λ_*y*_ = 3.1 µm (Figure 3e), and one photoalignment pattern has Λ_*x*_ = 3.1 µm and Λ_*y*_ = 6.65 µm (Figure 3f). The highest illumination dose *D*_2*avg*_ = 1920 mJ cm^−2^ in the second step (Figure 3a) gives rise to the formation of disinclination lines that connect defect points on the same substrate. The same type of configuration is formed over the whole area. The three lower illumination doses in the second step, Figure 3b–d, on the other hand, give rise to a vertical defect configuration recognized by a periodic array of black dots in the microscope images without polarizers. With the given illumination in the first step and the value for a (see further), based on the theory for two‐step illumination [39], the defect is obtained when the condition in Equation (4) is satisfied, which occurs for the second illumination dose *D*_2_ = 341 mJ cm^−2^ (see also Figure S1). The average illumination dose in the second step influences the defect spacing along the *x*‐axis, as will be discussed further. The presented results in Figure 3b–d, and also the result in Figure 3e with Λ_*x*_ = 6.65 µm and Λ_*y*_ = 3.1 µm, shows the same type of defect grid over the whole area. However, the experimental results with Λ_*x*_ = 3.1 µm and Λ_*y*_ = 6.65 µm (Figure 3f) show that it becomes more difficult to reliably stabilize the same type of defect configuration over large areas. Due to the very short spacing between the neighboring defects along the *x*‐axis, in some areas rewiring of disclination lines appears. In this case, disclinations similar to the ones in Figure 3a are also formed in some areas (connecting defect points on the same substrate). Remark that for larger distances from the center of the illumination, similar to the diameter of the Gaussian beams, the alignment patterns deteriorate. This can be mitigated by using a larger beam expander.

## 4 Simulations

To estimate the director configuration in the volume of the cell, numerical simulations are performed, making use of the double illumination theory to describe the surface alignment. In the simulations presented in this work, a cell thickness *t* of 1.4 µm is used, together with a pattern period Λ_*x*_ = Λ_*y*_ = 6.65 µm and illumination dose *D*_1_ = 1920 mJ cm^−2^, compatible with the experimental conditions. Four different values for *D*_2*avg*_ have been simulated, corresponding to 1920 , 960 , 630 , and 320 mJ cm^−2^. To obtain correspondence with the experimentally observed behavior, the value for the sensitivity parameter *a* in the double illumination theory is fixed to *a* = 0.002 cm^2^ mJ^−1^. A periodic unit cell with Λ_*x*_ by Λ_*y*_ dimension is simulated (making use of periodic boundary conditions along the *x*‐ and *y*‐ axis), and the surface anchoring is assumed to be strong. The pretilt angle is fixed to zero, and the azimuthal angle for the easy axis orientation is described by Equation (3). With the help of finite element *Q*‐tensor simulations, metastable director configurations with a local minimum in the Landau‐de Gennes free energy are found, as described in the Numerical Simulations Section.

**FIGURE 4:**
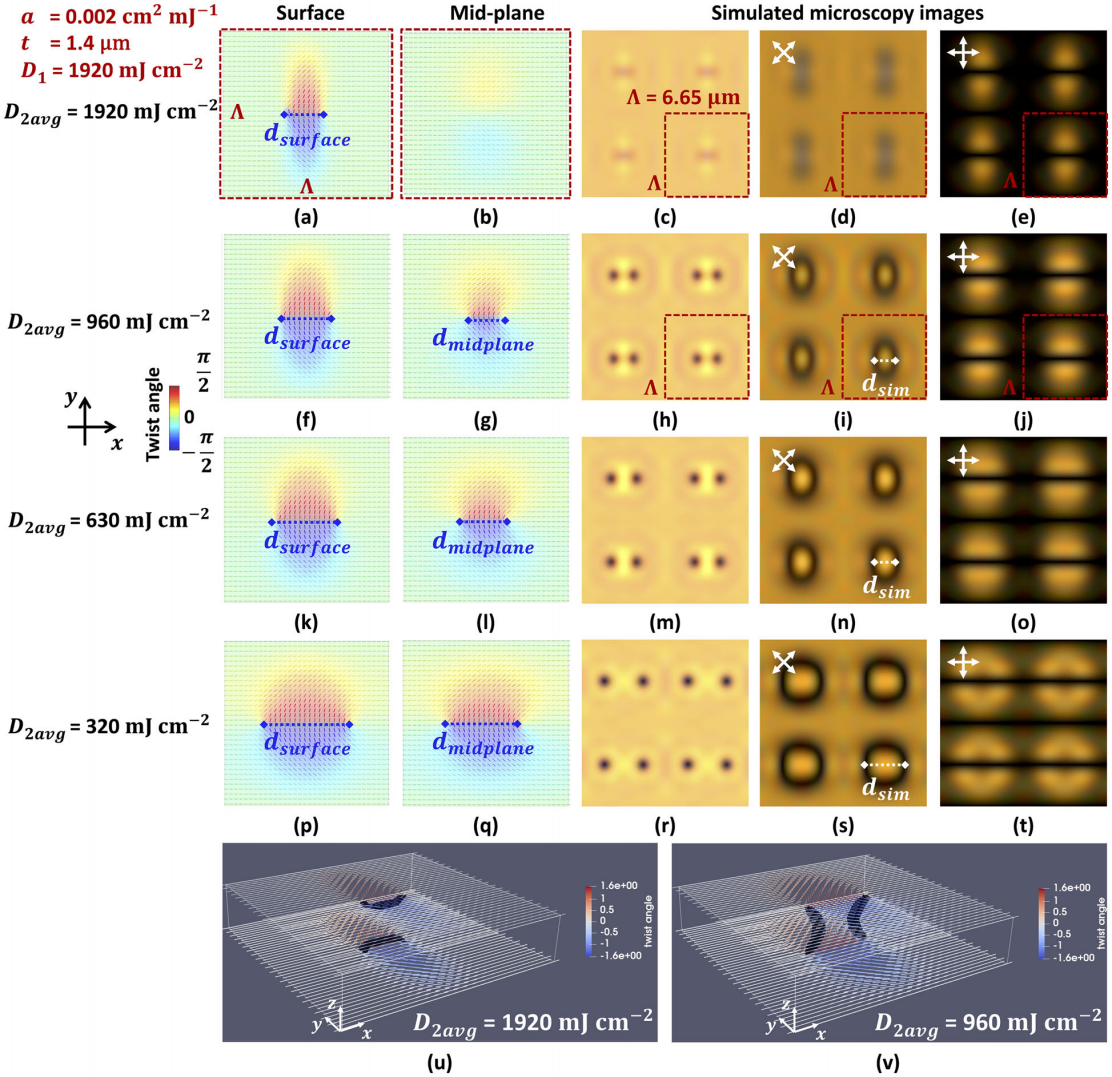
Director configurations in two dimensions and microscopy images simulated according to the double illumination theory. The parameter *a* is fixed at 0.002 cm^2^ mJ^−1^, the simulated cell thickness *t* is 1.4 µm, the pattern period Λ (in the *x*‐ and *y*‐direction) is 6.65 µm and the illumination dose in step I *D*_1_ is fixed at 1920 mJ cm^−2^. The director alignment in a Λ by Λ periodic unit cell is shown on the substrate plane (a,f,k,p) and in the mid‐plane of the cell $z=\frac{t}{2}$ (b,g,l,q). Transmissive microscopy images of a 2Λ by 2Λ area are given without polarizers (c,h,m,r) and with crossed polarizers (d,e,i,j,n,o,s,t). The second illumination dose *D*_2*avg*_ is 1920 mJ cm^−2^ in (a–e), 960 mJ cm^−2^ in (f–j), 630 mJ cm^−2^ in (k–o) and 320 mJ cm^−2^ in (p–t). 3D view of director configuration in the bulk with horizontal (u) and vertical (v) disclination lines marked with a dark color (indicating the region with reduced order parameter *S*  <  0.35). *D*_2*avg*_ is 1920 mJ cm^−2^ in (u) (corresponding to the results in (a–e)) and *D*_2*avg*_ is 960 mJ cm^−2^ in (v) (corresponding to the results in (f–j)).

The surface alignment patterns calculated based on the double‐illumination theory, for the four different doses in the second illumination step, are shown in Figure 4a,f,k,p. *D*_2*avg*_ is respectively 1920 , 960 , 630 , and 320 mJ cm^−2^ from top to bottom. The corresponding director configuration in the mid‐plane ($z=\frac{t}{2}$) after free energy minimization is shown in Figure 4b,g,l,q. The color represents the twist angle of the directors with respect to the *x*‐axis from $-\frac{\pi }{2}$ to $\frac{\pi }{2}$, with a green color indicating a LC director orientation along the *x*‐axis and a dark blue or red color indicating director alignment along the *y*‐axis. Note that the tilt angle remains practically constant, with the director being parallel to the substrate planes in the cell volume.

The combined effect of the first and second illumination step leads to a 2D surface alignment pattern (Figure 4a,f,k,p) with one +1/2 defect point and one ‐1/2 defect point per Λ_*x*_ by Λ_*y*_ unit cell. The larger the dose in the second illumination step, the larger the region with alignment close to the *x*‐orientation becomes and the smaller the horizontal distance between the +1/2 and ‐1/2 defects. The variation of *d_surface_* as a function of the illumination dose *D*_2*avg*_ is described by Equation (6) and is visible in Figure 4. The mid‐plane director configurations shown in Figure 4g,l,q, demonstrate that in these cases, half‐integer disclination lines propagate through the layer roughly along the *z*‐axis, connecting the surface defect points at the top and bottom substrates. However, even though the cell thickness is only 1.4 µm, the director configuration in the volume (Figure 4b,g,l,q) of the layer substantially deviates from the alignment pattern at the top and bottom substrate (Figure 4a,f,k,p). This can be verified by looking at the 3D Figure 4v that visualizes the trajectory of the disclination lines, by darkening the area with reduced order parameter (*S* <  0.35). It can be seen in Figure 4v that the roughly vertical disclination lines are bent, attracting each other and being closest to each other in the mid‐plane ($z=\frac{t}{2}$). The spacing in the mid‐plane is called *d_midplane_*. The attraction of the disclination lines in the bulk also explains why the roughly vertical disclination lines disappear when the dose *D*_2*avg*_ in the second illumination step is increased. When the defect spacing *d_surface_* at the surface becomes too small, the half integer disclination lines in the bulk rewire, forming disclination lines connecting the +1/2 and −1/2 surface defect points at the same substrate, as can be seen in Figure 4u. This explains the absence of singularities in Figure 4b: the mid‐plane director configuration in this case becomes roughly uniform, with a predominant alignment along the *x*‐axis.

Based on the simulated results for the bulk director configuration, optical simulations are performed to enable a comparison with the microscopy observations in Figure 2 and to confirm the validity of the director simulations. The details for the optical simulations are provided in the Numerical Simulations section. The simulated microscopy images are shown in Figure 4 for a 2Λ_*x*_ by 2Λ_*y*_ area and for three different orientations of the polarizers (no polarizers, crossed polarizers along the diagonals, and crossed polarizers along the *x*‐ and *y*‐ axis respectively). For the highest dose in step II *D*_2*avg*_ = 1920 mJ cm^−2^, the optical transmission without crossed polarizer in Figure 4c shows a 2 × 2 matrix with four slightly darker lines. The POM images with crossed polarizers along the diagonal and anti‐diagonal (Figure 4d), show four darker regions elongated along the *y*‐axis. When the average dose in step II *D*_2*avg*_ is decreased to 960 , 630 , or 320 mJ cm^−2^, the microscopy images without polarizers show four pairs of black dots in a matrix, as in Figure 4h,m,r. Each Λ_*x*_ by Λ_*y*_ unit cell gives rise to a pair of black dots, with the position of a black dot roughly corresponding to the position of a half‐integer disclination line running from the top to the bottom substrate. When adding crossed polarizers along the *xy*‐diagonal and anti‐diagonal in the microscope, four dark elliptic structures are observed in a bright surrounding area (Figure 4i,n,s). The width in the *x*‐ direction is denoted as *d_sim_* and will be used to compare with experimental measurements. When rotating the crossed polarizers over 45° (so that they are aligned along the *x*‐ and *y*‐ axis), the background changes to dark, and bright arch‐like regions are seen in Figure 4j,o,t. Remark that although these figures look rather similar to Figure 4e, for the configuration with horizontal disclination lines, the figures without polarizers (Figure 4c,h,m,r) or with another crossed polarizer orientation (Figure 4d,i,n,s) allow to clearly distinguish vertical and horizontal bulk disclination lines.

## 5 Discussion

The overall good agreement between the experimental results of Figure 2 and the numerical simulations of Figure 4, using only a few fitting constants, indicates that the double illumination theory for the alignment at the surface and the ansatz for the initial director distribution are valid approximations. In both the experiments and simulations, a high dose in the second illumination step results in the dominant formation of horizontal disclination lines, connecting two neighboring defects at the same substrate (Figures 2a–e, 3a, and 4a–e). The formation of these horizontal disclination lines is linked to the narrow spacing between the surface defect points and the small value of *d_surface_*. The narrow defect spacing can be induced by a high dose in the second illumination step, but a somewhat similar effect is obtained by decreasing the alignment period in the *x*‐ direction Λ_*x*_, explaining why some horizontal disclination lines are also observed in Figure 3f. A smaller dose in the second illumination step (or a larger period in the *x*‐direction Λ_*x*_) gives rise to a larger defect spacing at the surface *d_surface_* and the predominant formation of vertical disclination lines, connecting two defect points at opposing substrates (Figure 2f–t and 4f–t). The difference in total free energy between horizontal and vertical disclination lines depends on *d_surface_* and on the layer thickness, with vertical disclination lines being favored in thinner cells. The cell in our experiments (*t* = 1.4 µm) can stabilize a grid of vertical disclination lines even when the lateral spacing is in the order of only one to two micrometer.

Figure 2 (experimental) and Figure 4 (simulations) indicate that the distance between the −1/2 and +1/2 defects points decreases as a function of the illumination dose in step II *D*_2*avg*_.

**FIGURE 5:**
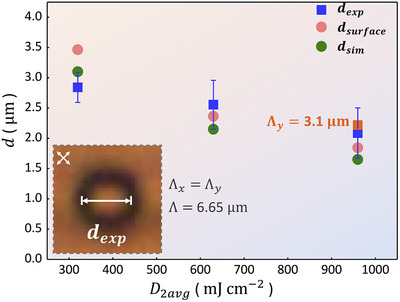
Defect spacing *d* as a function of the second illumination dose *D*_2*avg*_. Inset image: microscope image of one period Λ_*x*_ = Λ_*y*_ = 6.65 µm indicating how the experimental distance *d_exp_* is determined (white double arrow). Blue squares: *d_exp_* from microscope measurements. Red square: experimental value for the case that Λ_*y*_ is 3.1 µm. Green circles: *d_sim_* obtained from images based on numerical simulations, as in Figure 4. Red circles: distance *d_surface_* according to Equation (6) and to *a* = 0.002 cm^2^ mJ^−1^.

The distance *d_exp_* between two neighboring defects is determined by measuring the width of the dark ring along the *x*‐axis in the POM images for crossed polarizers along *xy*‐diagonal and antidiagonal, as illustrated in the inset in Figure 5. The blue squares represent the average value of the measured distance *d_exp_* from Figure 3, with the error bar showing the standard deviation (150 defect distances were measured). The values of *d_sim_* (green circles in Figure 5) are determined in a similar way, using the simulated microscope images for crossed polarizers along the *xy*‐diagonals as shown in Figure 4. The distance *d_surface_* between the two defects at the surface (red circles) is estimated from Equation (6). In each case, the distance decreases for increasing value of *D*_2*avg*_. The green circles, representing the distance estimated from the optical simulations *d_sim_*, are systematically below the red circles, representing *d_surface_*. This is expected, because the vertical disclination lines are attracting each other and their average distance is smaller than their distance at the surface. There is some deviation between the green circles (obtained by simulations) and the blue squares (obtained experimentally), but the trend is the same, and the deviations are in the order of the experimental variability. This confirms that the multiple‐illumination theory provides an adequate description for the surface alignment pattern [39]. Figure 5 demonstrates that a defect spacing of 2 µm can be obtained by using the double illumination interference photoalignment procedure. The image in Figure 3f indicates that defect spacings below 2 µm can be achieved, because the period in the *x*‐direction is only 3.1 µm. An analysis of the microscope image (Figure S2) reveals that the smallest experimental distance between two disclinations lines connecting opposite substrates is 1.25 µm.

**FIGURE 6:**
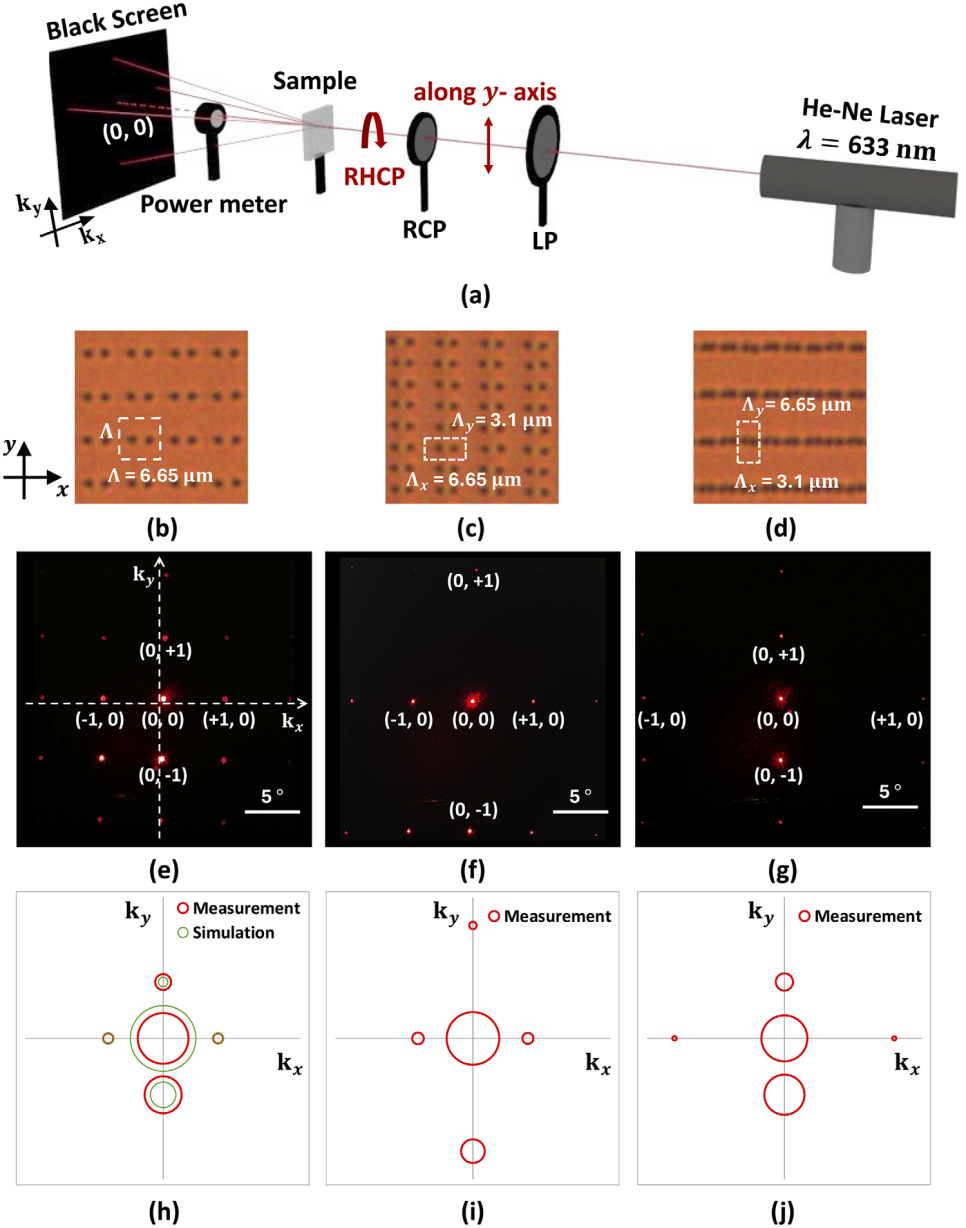
Diffraction of laser light incident on periodic structures with vertical disclination lines. (a) optical set‐up with a He‐Ne laser (Λ = 633 nm) and right‐handed circularly polarized incident light. (b–d) Microscopy images of arrays of disclination lines. (e–g) Images of diffraction patterns for right‐handed circularly polarized (RHCP) light. (h–j) Measured (red) and simulated (green) relative intensities of five diffraction orders, proportional with the area of the circle.

A highly periodic director pattern with a high density of disclination lines over a large area yields high quality large‐angle diffraction. The optical setup of Figure 6a uses a He‐Ne laser (wavelength 633 nm, beam diameter 1 mm), a linear polarizer (LP), a quarter wave plate, and a dark screen. The LP transmits vertically polarized light. A quarter wave plate is added to obtain right‐handed circularly polarized light (RHCP). The diffraction orders with the respective k‐vectors are detected on the dark screen. Figure 6e–g shows the diffraction patterns recorded by a camera for the patterns in Figure 6b–d. The fact that the spots are small and the background is dark indicates the excellent periodicity of the structure. The observed diffraction angle for the period of 6.65 µm is 5.6 degrees. A power meter is used to measure the incident power *P_total_* and the power of the diffraction orders $P_{\left(DO_{x},DO_{y}\right)}$. The diffraction efficiencies are calculated as ratios $\eta =P_{\left(DO_{x},DO_{y}\right)}/P_{total}\;$ and shown in Figure 5h–j, with the area of the circle proportional to the efficiency. Devices (b) and (c) have similar zero order transmittance (42% and 45% in the (0,0) order), while device (c) with Λ_*x*_ = 3.1 µm has a lower zero order transmission (35%). Note that the power in the orders (+1, 0) and (−1, 0) are similar, while the power in the (0, −1) order (23%, 10%, and 27%) is much larger than that in the (0, +1) order (5%, 1%, and 5%). This is reminiscent of the Pancharatnam‐Berry phase created by the rotating director during the first illumination step [21]. A similar asymmetry is reproduced in the simulated diffraction pattern shown in Figure 5h.

The experiments show that two‐step interference photoalignment can, without focusing requirements, realize grids of +1/2 and −1/2 defects with distances below 2 µm, which is ten times smaller than for grids based on the projection of a pixelated modulator. Between the defects, the azimuthal angle pattern is continuous, without effects due to pixelization. By using laser beams with a larger diameter, the total area of the grating can be scaled up, while increasing the angle between the two interfering beams may further scale down the unit cell. The produced photoalignment patterns have been stable for an extended period of time (several months), but to further improve the long‐term stability and avoid erasure by intense blue or UV light, covering the alignment layer with a thin polymerizable LC layer could be considered. Overall, these grids offer multiple options for interconnecting defects with robust disclination lines.

## 6 Conclusion

In conclusion, we have used the method of two‐step interference illumination to successfully realize a 2D array of defects and disclination lines in thin nematic LC cells. The first step uses interference of circularly polarized laser beams with opposite handedness to achieve a rotating director pattern. The second illumination partially overwrites this pattern by illumination with an interference pattern with intensity modulation. For appropriate illumination doses, a +1/2 and a −1/2 surface defect occur in each unit cell of the director pattern, with the distance between them determined by the illumination intensities. Each surface defect is connected through a disclination line in the bulk with another surface defect, either on the same substrate or on the opposite substrate. The distance between two neighboring vertical disclination lines can be in the order of 1 µm in a thin cell. The experimental results are supported by numerical simulations based on a theoretical model for multi‐step illumination to determine the director at the surface, the minimization of Landau‐de Gennes free energy to determine the director distribution in the bulk, and a beam propagation method to determine the transmission image. As the method is based on interference, it has the capacity to achieve high disclination line densities, with spacing similar to the wavelength of light, effectively creating soft, topologically protected metasurfaces, without effects of pixelization. The created structures can be used as reconfigurable templates for photonic devices to achieve large angle diffraction, beam shaping with large numerical aperture, or bistable smart windows.

## 7 Experimental Section

### 7.1 LC Cell Preparation

The LC cell consists of two pretreated glass substrates. The cleaned substrates were subjected to UV ozone exposure at 90°C for 15 min. The photosensitive chemical Brilliant Yellow (Sigma–Aldrich) was dissolved in Dimethylformamide (DMF) with a concentration of 0.5 wt.%. and deposited onto the glass substrates by a spin‐coating process. The spinning was at a speed of 3000 rpm for 30 s followed by a baking step at 90°C for 5 min. Two pretreated substrates were glued together along the edges with a glue (NOA 81) that contains spherical spacers with a diameter of 1.6 µm. The thickness of the LC cell was measured by a spectrophotometer and was found to be around 1.4 µm.

### 7.2 Two‐Step Interference

The empty LC cell was fixed on an interference set‐up consisting of a series of optical components. A continuous wave UV laser emitting at λ = 355 nm (Coherent, Genesis CX SLM, 100 mW) was used. The laser beam passed through a beam expander and a polarization beam splitter (PBS) that split the incident light into TE‐ and TM‐ polarizations. The relative intensities of the two beams after the PBS were modulated by a half waveplate (HWP 1) after the laser. In this way, an interference set‐up with the same polarization and intensity in both beams was obtained.

In the first step, two additional quarter waveplates (QWP) with fast axis at + / −  45°, respectively, were used to obtain right‐handed circularly polarized light (RHCP) and left‐handed circularly polarized light (LHCP) in two arms. A periodic pattern with rotating easy axis variation was obtained in the BY layer. In the second irradiation step, two QWPs were removed and second HWP was added. In the meantime, the cell was rotated over − 90° (Figure 1). An interference set up with TE polarization partially rewrites the pattern obtained after the first step. The periodicity of the resulting photoalignment pattern depends on the angle between two beams, which is governed by the interference formula $\Lambda =\frac{\lambda }{2\sin\theta }$, with θ the half opening angle between the two beams. The average illumination dose in step II *D*_2*avg*_ was varied experimentally by adjusting the time of the illumination (while keeping the intensity fixed).

After photo‐patterning, nematic LC (E7) was injected into the cell above the isotropic temperature at 80°C. The alignment of the LC was characterized at room temperature, with the help of a polarizing optical microscope (Nikon Eclipse LV100POL).

### 7.3 Numerical Simulations

Finite element *Q*‐tensor simulations are used to simulate the director configuration in the bulk of the layer with photopatterned anchoring substrates [40, 41]. The use of a *Q*‐tensor for the LC representation allows to simulate disclination lines, with a reduced order parameter at the core and a finite energy contribution. The double illumination theory [39] is used to describe the surface anchoring pattern, and strong anchoring conditions with a zero pretilt angle are applied. The cell thickness is fixed to 1.4 µm, and a Λ_*x*_ × Λ_*y*_ =  6.65 µm  ×  6.65 µm  unit cell is simulated, with periodic boundary conditions being applied along the *x*‐ and *y*‐direction. To find metastable solutions for the director configuration, the Landau‐de Gennes free energy is minimized starting from a well‐chosen initial condition. The free energy functional contains contributions from the elastic distortion energy, the surface energy, and the thermotropic (or bulk) energy. The elastic constants for the LC material E7 are taken into account (*K*_11_ = 11.1 *pN*, *K*_22_ = 6.5 *pN*, *K*_33_ = 17.1 *pN*). The bulk thermotropic coefficients are based on the ones measured for 5CB at a reduced temperature of −2°C (A = −174 N m^−2^, B = −2120 N m^−2^, C = 1740 N m^−2^), giving rise to an equilibrium order parameter of 0.5 [42]. These values are reduced 1000 times, artificially rescaling the core size of the defects but also allowing for much faster computations [43]. The mesh density is sufficiently high to ensure the mobility of disclination lines. The initial condition that is used in the presented simulations (Figure 3) is invariant in the *z*‐direction, extending the surface anchoring conditions over the whole volume. This gives rise to a starting configuration containing a set of two straight disclination lines (along the *z*‐axis) in every unit cell. The spacing along the *x*‐axis between these disclination lines is *d_surface_*, as found by the double illumination photoalignment theory (Equation 6). During the free energy minimization, the vertical disclination lines start to bend (Figure 3g,l,q,v) and can rewire, forming horizontal disclination lines, for the highest illumination dose *D*_2*avg*_ in the second alignment step (Figure 3b,u).

To simulate the observed behavior in the microscope, the open‐source optical simulation package Nemaktis (https://github.com/warthan07/Nemaktis) is used [44]. This tool makes use of a generalized beam propagation method to calculate the electromagnetic field distribution throughout the LC layer. The resulting director configuration obtained from the Q‐tensor simulations is interpolated on a regular grid and used for the LC layer description in Nemaktis. Light is propagated through the layer, taking into account a contribution from the phase evolution, a diffraction operator, and a walk‐off operator. The measured refractive indices for E7 are used in the simulations, ($n_{o}=1.49669+\frac{0.00785}{\lambda^{2}}+\frac{0.00026}{\lambda^{4}}$, $n_{e}=1.67906+\frac{0.01546}{\lambda^{2}}+\frac{0.001663}{\lambda^{4}}$) and 15 equally spaced wavelengths between 400 nm and 750 nm are used to create color images. To do so, CIE illuminant A is taken into account, and to better reproduce the experimental measurements, the focusing optics in the microscope is also accounted for (using numerical aperture 0.3).

To generate the simulated diffraction pattern in Figure 5h, the optical simulation starts from the simulated director configuration (Figure 3v) and considers the polarization (circular) and the incident laser wavelength (633 nm) for the diffraction experiments. Based on the near‐field transmission, a 2D discrete Fourier transform is performed to find the far‐field diffraction pattern.

## Conflicts of Interest

The authors declare no conflicts of interest.

## Supporting information




**Supporting File**: adma72607‐sup‐0001‐SuppMat.docx.

## Data Availability

The data that support the findings of this study are available from the corresponding author upon reasonable request.
