# Supplementary material for: High‐Density Topological Defect Array by Two‐Step Interference Photoalignment
Source: Adv Mater. 2026 Feb 19;38(17):e72607. doi: 10.1002/adma.72607 (PMC13003911; doi:10.1002/adma.72607)
Supplement: Supplementary file 1 — Supporting File: adma72607‐sup‐0001‐SuppMat.docx. [file ADMA-38-e72607-s001.docx]

Supporting Information

**High-density Topological Defect Array by Two-step Interference Photoalignment**

*Sunqian Liu, Inge Nys, Kristiaan Neyts**


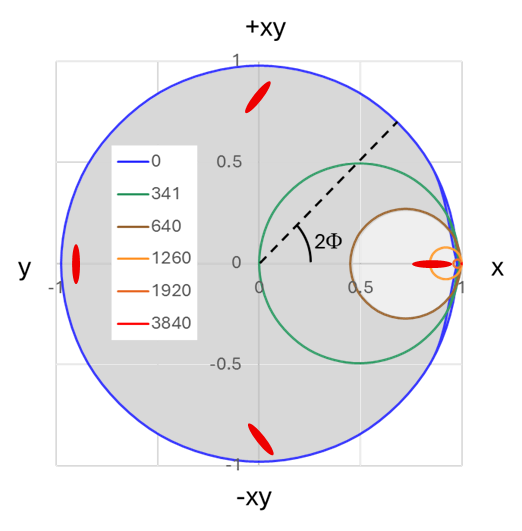


**Figure S1.** Parameter space of the alignment vector $\mathbf{n}$ with azimuth angle 2$\Phi$ (twice that of the LC azimuthal angle). The six curves are for different intensities $D_{2}$ of the second illumination step (the intensity of first illumination is fixed at 1920 $mJ cm^{-2}$). Different points on the same curve are for different values of the *y*-coordinate (different azimuthal angle). The green curve is for $D_{2}$= 341 $mJ cm^{-2}$, this curve contains the origin, a defect point, where the director orientation is not defined. The red ellipses illustrate the director orientation. The dark gray area between the blue and the brown circle indicates the possible alignment vectors when the second illumination intensity is between 0 and 640 $mJ cm^{-2}$, corresponding to the variation for $D_{2avg}$= 320 $mJ cm^{-2}$.

This paragraph provides a more detailed calculation of the photoalignment due to two step illumination, based on the theory of ref [1] and illustration in **Figure S1**.

In the theory for photoalignment under influence of two-step illumination (see [1]):

- the illumination in each step is characterized by the in-plane component of the Stokes polarization vector $\boldsymbol{s}$ and the dose of the illumination $D=It$ (product of intensity and time of the illumination);
- the alignment vector $\mathbf{n}$ describes the azimuthal angle of the preferred director and the strength of the alignment.

The time variation of the alignment vector, with initial value $\mathbf{n}_{0}$, under influence of the illumination with polarization vector ***s*** is given by:

$$\begin{aligned} \mathbf{n}\left( t \right)= -\mathbf{s}\left( 1-\exp\left( -aIt \right) \right)+\mathbf{n}_{0}\exp\left( -aIt \right)\boldsymbol{.\#} \end{aligned}$$

After two illumination steps, the alignment vector becomes, after integration (with the index referring to the sequence of the illumination step):

$$\begin{aligned} \mathbf{n}= -\mathbf{s}_{2}\left( 1-\exp\left( -aD_{2} \right) \right)-\mathbf{s}_{1}\left( 1-\exp\left( -aD_{1} \right) \right)\exp\left( -aD_{2} \right)\boldsymbol{.\#} \end{aligned}$$

In our experiments we used the following parameters:

- Polarization vector $\boldsymbol{s}\left( y \right)$ in step I due to azimuth $\varphi_{1}$: ($\cos2\varphi_{1},sin2\varphi_{1}$) with

$$\varphi_{1}\left( y \right)=\frac{\pi}{2}-\frac{\pi}{\Lambda_{y}}y$$

- Illumination dose in step I: $D_{1}=1920 mJ cm^{-2}$
- Polarization vector $\boldsymbol{s}$ in step II with azimuth angle $\varphi_{2}=\frac{\pi}{2}$ ($y$polarization): ($-1,0$)
- Variable illumination dose in step II: $D_{2}\left( x \right)=2D_{2avg}\cdot{cos}^{2}\left( \frac{\pi}{\Lambda_{x}}x \right)$
- The parameter *a* is chosen as $0.002 cm^{2}{mJ}^{-1}$ to fit the experiments.

The figure shows the alignment vector $\mathbf{n}$ for 6 different intensities of the second illumination dose (6 curves, for $D_{2}$= 0 to 3840 $mJ cm^{-2}$), where the variable polarization vector $\mathbf{s}$during the first illumination step is a parameter. The green curve, for the value $D_{2}$= 341 $mJ cm^{-2}$ goes through the origin, which means that the alignment is not determined, leading to a defect).

Note that the azimuth of the preferred LC alignment $\Phi$, is half of the azimuth of the alignment vector $\mathbf{n}$ in the diagram ($2\Phi$). The azimuthal direction of the LC alignment is indicated in the diagram by red ellipses.

The dark grey area in the diagram illustrates the possible locations for the vector $\mathbf{n}$ (varying as a function of the $x$,$y$ coordinates), when the second illumination step has $D_{2avg}$= 320 $mJ cm^{-2}$ and $D_{2}$varies between 0 and 640 $mJ cm^{-2}$_._


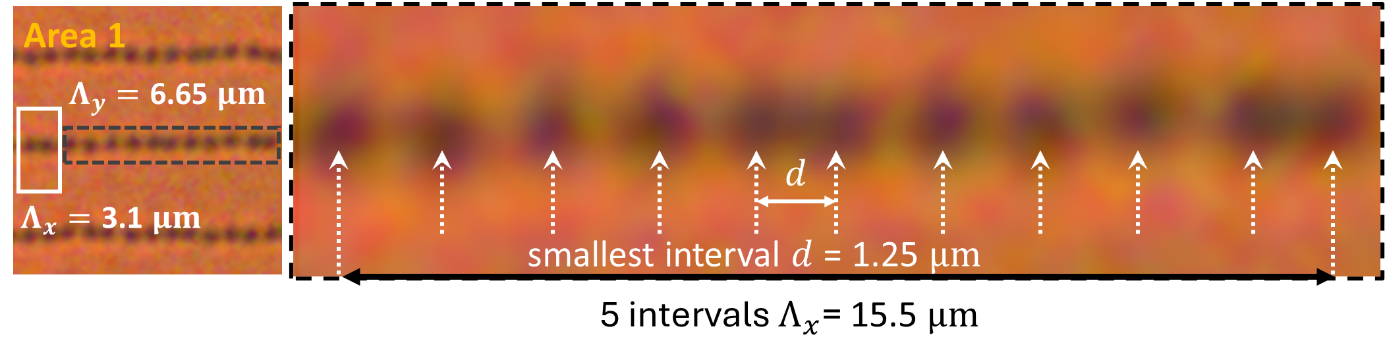


**Figure S2.** Magnification of a sequence of $z$-oriented disclination lines in Figure 4f, observed by optical microscopy (region in the dashed line rectangle). The average distance between two disclination lines is $\frac{\Lambda_{x}}{\text{2}}=\text{1.55} \mu m$, but there is some variability, with the smallest observed distance being 1.25 $\mu m$.

To estimate the distance between defects at the surface, area 1 in Figure 4f is investigated in more detail in **Figure S2**, because in this experiment the period in the horizontal direction is only 3.1 $\mu m$. The average distance between the disclination lines is 1.55 $\mu m$, but there is some variability, and the smallest observed separation is 1.25 $\mu m$. This illustrates that in nematic liquid crystal, defects and disclinations lines connecting opposite substrates in photoaligned devices, can have a separation of only 1.25 $\mu m$.

**References**

[1] B. Berteloot, I. Nys, S. Liu, K. Neyts, *ACS Appl. Opt. Mater.* **2023**, *2*, 1295.
